# New insights into N6-methyladenosine in hepatocellular carcinoma immunotherapy

**DOI:** 10.3389/fimmu.2025.1533940

**Published:** 2025-01-22

**Authors:** Mengran Li, Hu Tian, Yanshuang Zhuang, Zili Zhang

**Affiliations:** ^1^ Department of Science and Technology, Taizhou Affiliated Hospital of Nanjing University of Chinese Medicine, Nanjing University of Chinese Medicine, Taizhou, Jiangsu, China; ^2^ School of Pharmacy, Nanjing University of Chinese Medicine, Nanjing, China

**Keywords:** hepatocellular carcinoma, RNA N6-methyladenosine modification, immunotherapy, therapeutic target, clinical treatment

## Abstract

N6-methylation is a modification in which a methyl group is added to the adenine base of a nucleotide. This modification is crucial for controlling important functions that are vital for gene expression, including mRNA splicing, stability, and translation. Due to its intricate participation in both normal cellular processes and the course of disease, as well as its critical role in determining cell fate, N6-methyladenosine (m^6^A) alteration has recently attracted a lot of interest. The formation and progression of many diseases, especially cancer, can be attributed to dysregulated m^6^A alteration, which can cause disturbances in a variety of cellular functions, such as immunological responses, cell proliferation, and differentiation. In this study, we examine how m^6^A dysregulation affects hepatocellular carcinoma (HCC), with a particular emphasis on how it contributes to immunological evasion and carcinogenesis. We also investigate its potential as a novel therapeutic target, providing new perspectives on potential therapeutic approaches meant to enhance clinical results for patients with HCC.

## 1 Introduction

In eukaryotes, RNA modifications occur in a variety of forms, including N6-methyladenosine (m^6^A), 5-methylcytosine (m^5^C), and N7-methylguanosine (m^7^G) (1, 2). The processing and function of RNA are greatly impacted by these chemical alterations. In eukaryotic mRNA, m^6^A is the most prevalent reversible chemical alteration that is controlled by the collaborative efforts of “writers,” “erasers,” and “readers.” RNA is modified by writers by adding methyl groups, which reader proteins then recognize. Through this interaction, the altered RNA is able to carry out a number of vital tasks that eventually affect gene expression, such as splicing, stability regulation, translation, and destruction (3). M^6^A erasers can erase methyl groups. Recent research has demonstrated that m^6^A has a role in the progression of several disorders, including ovarian cancer (4), colorectal cancer (5), pancreatic cancer (6), and liver cancer (7). During tumor development, m^6^A not only promotes the expression of tumor-related genes, driving tumor progression, but also exerts therapeutic effects during cancer treatment by modifying the targets of radiation therapy.

Liver cancer is one of the leading causes of cancer-related deaths worldwide and is the only type of cancer that has shown a consistent annual increase in incidence (8). Various factors contribute to its development, including hepatitis B, hepatitis C, obesity, alcoholic fatty liver disease, and iron overload. Liver cancer can be treated with surgical resection if detected early. However, the majority of patients are diagnosed at an advanced stage. The standard clinical treatment, sorafenib, is associated with drug resistance and significant toxic side effects during prolonged use. As a result, developing effective therapies for liver cancer remains a pressing need. As an essential immune organ, the liver contains a wide variety of immune cells, including Kupffer cells (KCs), natural killer (NK) cells, natural killer T cells, helper innate lymphoid cells (ILCs), dendritic cells (DCs), macrophages, and granulocytes. Together, these cells play a key role in preserving the body’s homeostasis (9). Chronic liver disease disrupts the balance of homeostasis, with immune cells playing dual roles in either promoting or inhibiting liver cancer immunotherapy. Gaining insight into the mechanisms by which these immune cells influence liver cancer and developing strategies to target them for immunotherapy could provide valuable perspectives for advancing clinical treatment.

## 2 N6-methyladenosine

### 2.1 m^6^A writers

M^6^A methylation is the process by which some methyltransferases catalyze the addition of a methyl group to the nitrogen-6 (N6) position of adenosine (10). These include methyltransferase-like 3 (Mettl3), Mettl 14, Mettl 16, Wilms tumor 1-associated protein (WTAP), RNA-binding proteins 15/15B, and the human zinc finger protein domain 13. The first enzyme found to cause m^6^A methylation among them was Mettl3 (11). S-adenosylmethionine (SAM) serves as the methyl donor for Mettl3 and is crucial for its enzymatic activity (12). Mettl14, a Mettl3 homolog, lacks catalytic activity but functions as an RNA-binding scaffold. Mettl14 improves Mettl3’s catalytic function by enhancing its interaction with target RNA (13). Mettl14 is critical for hepatocyte renewal, differentiation, and tumor growth. Furthermore, WTAP forms a complex with Mettl3 and Mettl14, enhancing their ability to bind target RNA and contributing to embryonic differentiation (14). In recent years, additional methyltransferases have been identified as key players in the RNA m^6^A methylation process. These include Mettl16, RNA-binding proteins 15/15B (RBM15/15B) (14), human zinc finger protein containing domain 13 (ZC3H13), and vir-like m^6^A methyltransferase-associated protein (VIRMA) (15).

### 2.2 m^6^A readers

M^6^A readers are the primary effectors of m^6^A methylation, functioning by recognizing methylated RNA bases. These proteins bind to m^6^A modification sites and regulate various RNA processes, including gene expression, splicing, export, and translation efficiency (16). The YT521-B homology (YTH) domain family consists of YTHDF1, YTHDF2, YTHDF3, YTHDC1, and YTHDC2 (17). The cytoplasm is home to the majority of them, including YTHDF1, YTHDF2, and YTHDF3, which mediate post-transcriptional changes. On the other hand, YTHDC1 and YTHDC2 are found in the nucleus and control nuclear export and RNA splicing. The heterogeneous nuclear ribonucleoprotein (HNRNP) family members, such as HNRNPA2B1, HNRNPC, HNRNPG, and the eukaryotic initiation factor 3 (eIF3), are also key readers of m^6^A methylation. Specifically, HNRNPA2B1 functions by activating downstream pathways of miRNA primers and regulating miRNA maturation (18). eIF3 proteins facilitate mRNA translation by binding to the m^6^A modification in the RNA 5’ untranslated region (UTR) (19). M^6^A methylation readers are crucial regulators of RNA function that regulate gene expression. Knowing how they work provides important information about the ways in which m^6^A methylation affects RNA biology.

### 2.3 m^6^A erasers

M^6^A “erasers” are enzymes that remove the methyl group from the N6 position of adenosine in RNA, effectively reversing the effects of m6A methylation (20). These enzymes are key in regulating RNA methylation dynamics and maintaining the methylation-demethylation equilibrium. The main m^6^A erasers discovered are fat mass and obesity-associated protein (FTO) and AlkB homolog 5 (ALKBH5) (21). FTO was first discovered to regulate body weight and metabolism, however it is also a m^6^A demethylase. It eliminates m^6^A tags from RNA, which affects its stability, splicing, and translation. FTO was discovered to function as a m^6^A demethylase after first being discovered to regulate body weight and metabolism. It affects RNA stability, splicing, and translation by eliminating m^6^A tags from RNA. Numerous physiological functions, including as energy homeostasis, brain development, and stress response, have been linked to FTO (22). Likewise, another important demethylase that selectively targets m^6^A alterations is ALKBH5. It plays a role in controlling nuclear export, stability, and RNA splicing. It has been demonstrated that ALKBH5 is essential for stem cell self-renewal and differentiation, and that its dysregulation can impact cellular reactions to external stressors and accelerate the course of disease (23). These erasers mediate a balance between m^6^A methylation and demethylation, which is crucial for regulating RNA activities like gene expression, RNA maturation, and destruction. Gaining knowledge about how m^6^A erasers work will help one better understand how RNA metabolism is regulated and how it affects various cellular functions as well as disease mechanisms.

## 3 The role of m^6^A methylation in liver cancer immunotherapy

With a high rate of recurrence, liver cancer is the most common and dangerous type of cancer. It still has a high incidence and fatality rate worldwide, and there are few and frequently ineffective therapeutic options (24). In addition, a number of other malignancies, such as colorectal, lung, and pancreatic tumors, can metastasize primarily to the liver. Recent years have seen modest advancements in immunotherapy for the liver, a vital immunological organ (25). Therefore, the development of successful immunotherapies for liver cancer and the improvement of patient prognosis dependent on an understanding of the underlying molecular pathways. Over the past few years, a number of studies have demonstrated a connection between drug resistance, tumor invasion, and metastasis and the aberrant expression of proteins involved in m^6^A alteration. According to these studies, m^6^A alterations control liver immunity and may have promotive or anticancer effects (26). As a result, m^6^A alteration might eventually emerge as a crucial therapeutic target for immunotherapy for liver cancer. The biological roles and possible mechanisms of proteins linked to m^6^A modification in controlling target and downstream immune-related genes during the development of hepatocellular carcinoma (HCC) will be thoroughly reviewed in the sections that follow, offering a theoretical foundation for the clinical management of HCC.

### 3.1 The role of m^6^A methylation writers in liver cancer immunotherapy

The relationship between m^6^A writers and liver cancer immunotherapy is multifaceted and highly significant. An analysis of HCC patients from the TCGA database, combining transcriptomic and clinical data through clustering, identified several m^6^A-related genes associated with HCC progression. A risk assessment model, developed based on these genes, revealed that patients with different risk scores exhibited distinct survival outcomes, variations in immune therapy biomarker expression, TP53 mutation rates, and differential sensitivity to chemotherapy and targeted therapies. This highlights the potential of m^6^A-related genes to serve as biomarkers for predicting prognosis and tailoring individualized treatment strategies for liver cancer. Furthermore, these genes are anticipated to play a critical role in advancing liver cancer therapy, particularly in elucidating the immune characteristics of HCC and improving therapeutic response prediction in the future (27).

In the context of liver cancer progression, single-cell RNA sequencing has illuminated the pivotal role of METTL3, a key m^6^A writer, in modulating the immune microenvironment. Pan et al. demonstrated that METTL3 significantly reduces the infiltration of granzyme B (GZMB^+^) and interferon-gamma-positive (IFN-γ^+^) CD8^+^ T cells during the development of liver cancer, thereby inhibiting tumor immunity and promoting immune evasion in HCC. This immune suppression facilitated by METTL3 impairs the tumor’s ability to be targeted by the immune system, contributing to its progression and resistance to immune therapies (28). Additionally, METTL3 has been shown to mediate the m^6^A modification of Sterol Regulatory Element-Binding Protein (SREBP) Cleavage-Activating Protein (SCAP) mRNA, enhancing its translation and leading to increased cholesterol synthesis. This metabolic alteration negatively impacts CD8^+^ T cell function in HCC cells, further exacerbating immune evasion. Given these insights, targeting METTL3 with specific inhibitors, such as STM2457, in combination with immune checkpoint inhibitors like anti-PD-1 therapy, offers a promising therapeutic strategy. This combination has the potential to reverse immune suppression, activate CD8^+^ T cells, and improve the efficacy of liver cancer immunotherapy, thereby providing a novel approach to overcoming immune evasion in HCC (29).

METTL14, a critical component of the m^6^A RNA methyltransferase complex, plays a pivotal role in regulating the initiation, progression, and modulation of the immune microenvironment in liver cancer. Through m^6^A modification, METTL14 influences various aspects of RNA metabolism, including mRNA splicing, stability, and translation efficiency. Its dysregulation has been closely linked to tumor growth, immune evasion, and resistance to immunotherapy in HCC (30, 31). In HCC, METTL14 enhances the expression of immune-suppressive molecules and facilitates the recruitment of immune-suppressive cells, such as regulatory T cells (Tregs) and myeloid-derived suppressor cells (MDSCs). This immune reprogramming weakens the antitumor activity of effector T cells and natural killer (NK) cells, thus promoting the immune evasion capabilities of liver cancer cells (32). One of the key mechanisms by which METTL14 exerts its effects is through the regulation of PD-L1 mRNA stability and translation via m^6^A modification. By increasing PD-L1 expression on the surface of liver cancer cells, METTL14 dampens the immune response by inhibiting the activation of effector T cells. This, in turn, reduces the effectiveness of immune checkpoint inhibitors, such as anti-PD-1 and anti-PD-L1 antibodies, highlighting METTL14’s central role in immune resistance in liver cancer (30, 31). Furthermore, METTL14 modulates the expression of various chemokines (e.g., CXCL1, CCL2), which promote the expansion of immune-suppressive cells within the tumor microenvironment. This contributes to an immunosuppressive milieu, further impairing the antitumor immune response. Targeting METTL14 represents a promising strategy for enhancing liver cancer immunotherapy. Inhibition of METTL14 activity or downregulation of its expression has been shown to decrease PD-L1 levels, restore T cell function, and reverse the immunosuppressive status of the tumor microenvironment. Additionally, combining METTL14 inhibitors with immune checkpoint inhibitors may overcome resistance to single-agent therapies, significantly improving therapeutic efficacy (33). Given its critical role in immune evasion and treatment resistance, METTL14 serves as an important therapeutic target. A more comprehensive understanding of the molecular mechanisms through which METTL14 modulates immune responses, in conjunction with the development of selective inhibitors, has the potential to uncover new therapeutic avenues, facilitating the advancement of more targeted and efficacious immunotherapy strategies for liver cancer (33).

METTL16, an RNA m^6^A methyltransferase, has recently emerged as a critical regulator of the immune microenvironment in HCC, influencing tumor progression and response to immunotherapy. This enzyme catalyzes the m^6^A modification on RNA molecules, including mRNA, lncRNA, and miRNA, modulating RNA stability, splicing, and translation. In the context of HCC, METTL16 plays a significant role in immune evasion by regulating key immune checkpoint molecules such as programmed cell death ligand 1 (PD-L1). PD-L1, when bound to its receptor PD-1 on T cells, suppresses immune responses and facilitates tumor immune escape. Mettl16 enhances PD-L1 expression by promoting the m^6^A modification of its mRNA, increasing its stability and subsequent translation. This regulation contributes to the establishment of an immunosuppressive microenvironment that facilitates HCC progression and resistance to immune checkpoint inhibitors (ICIs). Inhibition of Mettl16 has been shown to downregulate PD-L1 expression, enhancing T cell-mediated anti-tumor responses and improving the efficacy of immunotherapies such as PD-1/PD-L1 blockade (34). In addition to modulating immune checkpoints, Mettl16 influences the polarization of tumor-associated macrophages (TAMs), a key component of the immune infiltrate in solid tumors. TAMs can be polarized into either pro-inflammatory M1 or immune-suppressive M2 phenotypes, with the latter promoting tumor progression and immune suppression. Mettl16 drives M2 polarization by enhancing the stability and translation of mRNA transcripts encoding immunosuppressive cytokines, including IL-10 and TGF-β. These cytokines suppress effector T cell activity and further reinforce the immunosuppressive environment. Moreover, Mettl16 regulates other immune cell populations, including regulatory T cells (Tregs), which play a central role in immune tolerance and tumor immune evasion. By modulating the expression of cytokines and chemokines involved in Treg recruitment and activation, Mettl16 contributes to their accumulation in the tumor microenvironment, further exacerbating immune suppression. These findings underscore the critical role of Mettl16 in shaping the immune landscape of HCC and highlight its potential as a therapeutic target to overcome resistance to immunotherapy. Targeting Mettl16 could reprogram the immune microenvironment, reduce PD-L1 expression, alter macrophage polarization, and diminish Treg-mediated immune suppression, thereby enhancing the efficacy of immune-based treatments (35). In conclusion, METTL16 serves as a pivotal modulator of immune evasion in HCC, regulating immune checkpoints, macrophage polarization, and Treg function. By orchestrating these immune processes, Mettl16 contributes to the establishment of an immunosuppressive tumor microenvironment that hampers the effectiveness of immunotherapies. Targeting Mettl16 represents a promising strategy to enhance the efficacy of current immunotherapeutic approaches and to overcome resistance mechanisms in liver cancer. This epitranscriptomic regulator offers new avenues for improving clinical outcomes in patients with HCC, emphasizing the need for further investigation into its potential as a therapeutic target in cancer immunotherapy (36, 37).

The stability and site-specificity of m^6^A alterations are preserved by this essential regulatory member of the m^6^A methyltransferase complex. In recent years, WTAP has been identified as having a significant oncogenic role, particularly in HCC, where it is closely linked to the formation of the tumor immune microenvironment and the response to immunotherapy. WTAP can affect the expression of several immune-related genes, such as chemokines, inflammatory factors, and immunological checkpoint molecules, via controlling m^6^A alterations. This can alter tumor cells’ ability to evade the immune system and the efficacy of immunotherapy. First, WTAP significantly promotes the overexpression of immunological checkpoint molecules like PD-L1 by stabilizing their mRNA through m^6^A alterations. When PD-L1 is overexpressed, effector T cell function is inhibited, which increases tumor cells’ capacity for immune evasion and reduces the effectiveness of immune checkpoint inhibitors such anti-PD-1/PD-L1 antibodies (7). Additionally, WTAP controls the expression of chemokines (such CCL5 and CXCL10) that facilitate the recruitment of immunosuppressive cells into the tumor microenvironment, including regulatory T cells (Tregs) and myeloid-derived suppressor cells (MDSCs). Patients become less sensitive to immunotherapy as a result of this worsening of the immunosuppressive condition (31). Furthermore, WTAP contributes significantly to the development of liver cancer immunotherapy resistance. According to studies, WTAP stimulates signaling pathways such PI3K/AKT and Wnt/β-catenin through m^6^A alterations. These pathways not only encourage tumor cell migration and proliferation but also increase the tumor’s resistance to T cell-mediated immune destruction (38). The cytotoxicity of CD8^+^ T cells against tumor cells is markedly diminished in liver cancer patients with high WTAP expression. This is accompanied by increased expression of immune checkpoint molecules and decreased infiltration of anti-tumor immune cells. Furthermore, WTAP plays a key role in liver cancer resistance to immunotherapy. According to research, WTAP stimulates important signaling pathways such PI3K/AKT and Wnt/β-catenin through m^6^A alterations. These mechanisms enhance the tumor’s resistance to T cell-mediated immune destruction in addition to encouraging tumor cell migration and proliferation (38). While immune checkpoint molecules are expressed at higher levels and anti-tumor immune cell infiltration is decreased, CD8^+^ T cell-mediated cytotoxicity against tumor cells is considerably reduced in liver cancer patients with elevated WTAP expression. Through complex m^6^A regulation, WTAP plays a critical role in tumor microenvironment remodeling, resistance development, and immune evasion in liver cancer. In addition to delivering novel clinical solutions in the realm of cancer treatment, a better knowledge of the molecular mechanisms underlying WTAP and the creation of specific inhibitors against it may open up new avenues for precise immunotherapy in liver cancer.

Selectively identifying the m^6^A modification sites is the main function of this significant auxiliary protein of the m^6^A methyltransferase complex. It is essential for both the immune microenvironment’s control and the onset and spread of HCC. According to studies, VIRMA affects the m^6^A alteration of several immune-related genes, which controls the tumor cells’ resistance to immunotherapy and their capacity to avoid immune detection. Increased PD-L1 expression on the surface of tumor cells results from VIRMA’s enhancement of the m^6^A alteration of immune checkpoint molecules, including PD-L1 mRNA, which improves its stability and translation efficiency. Because effector T cell activity is suppressed, the tumor’s reaction to immune checkpoint inhibitors (such anti-PD-1/PD-L1 antibodies) is weakened (39). Further suppressing anti-tumor immune responses, VIRMA also controls the m^6^A modification levels of chemokines and inflammatory cytokines (like CXCL1 and IL-6), which encourages the growth of myeloid-derived suppressor cells (MDSCs) and regulatory T cells (Tregs) in the tumor microenvironment (39). Furthermore, the abnormal expression of VIRMA is closely associated with the resistance of liver cancer to immunotherapy. Several studies have shown that VIRMA activates signaling pathways such as PI3K/AKT and Wnt/β-catenin, which enhance tumor cell proliferation and resistance to T cell-mediated cytotoxicity (7). At the same time, VIRMA regulates genes related to metabolism through m6A modifications, promoting metabolic reprogramming in tumor cells, which enhances their survival ability in the immunosuppressive microenvironment. Targeting VIRMA presents a potential strategy to enhance the efficacy of liver cancer immunotherapy. By inhibiting VIRMA expression or blocking its m^6^A modification activity, the expression of PD-L1 and immune suppressive factors can be effectively reduced, restoring immune activity within the tumor microenvironment (40). Additionally, combining VIRMA inhibitors with immune checkpoint inhibitors could help overcome resistance, enhancing the anti-tumor effects of immunotherapy (41). Therefore, VIRMA affects liver cancer immune escape and treatment response via a variety of molecular pathways as a major regulator of m^6^A modification. Thorough research into its processes and the creation of specific inhibitors will greatly enhance clinical results and offer new approaches for precision immunotherapy in liver cancer.

RBM15 and RBM15B are critical members of the RNA-binding protein family, playing essential roles in regulating RNA splicing, stability, and translation. These proteins significantly influence immune evasion and the efficacy of immune therapies in liver cancer. Recent studies have highlighted their dual role in both the initiation and progression of liver cancer, as well as their involvement in reshaping the tumor immune microenvironment. The expression levels of RBM15 and RBM15B are closely linked to prognosis, immune evasion mechanisms, and resistance to immunotherapy in liver cancer patients. Specifically, these proteins modulate immune cell function within the tumor microenvironment, exerting immunosuppressive effects that hinder effective anti-tumor immune responses. RBM15 has been shown to promote the accumulation of Tregs and immune-suppressive M2 macrophages, both of which contribute to immune evasion by reducing the immune system’s ability to recognize and attack tumor cells. Conversely, RBM15B regulates the expression of key immune checkpoint molecules such as PD-1 and CTLA-4, thereby enhancing immune tolerance and further aiding tumor cells in escaping immune surveillance. These mechanisms play a crucial role not only in the promotion of immune evasion in liver cancer but also in the development of resistance to immune checkpoint inhibitors, such as PD-1/PD-L1 antibodies. As such, both RBM15 and RBM15B represent promising therapeutic targets for liver cancer immunotherapy. Inhibiting the expression of RBM15 and RBM15B has been shown to restore the immune system’s capacity to recognize and effectively target liver cancer cells, thus enhancing the therapeutic efficacy of immune therapies. Such interventions could provide a novel approach for overcoming resistance to immune checkpoint inhibitors, making RBM15 and RBM15B inhibition a potentially effective strategy to improve liver cancer treatment outcomes. These findings suggest that targeting RBM15 and RBM15B offers a promising avenue for enhancing immune responses and improving the overall effectiveness of liver cancer immunotherapy (42, 43).

### 3.2 The role of m6A methylation readers in liver cancer immunotherapy

Research has demonstrated that YTHDF1, a key m^6^A reader protein, plays a significant role in regulating the immunogenicity of liver cancer cells through its modulation of various tumor-associated genes (44). YTHDF1 specifically recognizes and binds to m^6^A modifications on RNA, influencing the stability and translation of target transcripts involved in immune evasion and tumor progression. By enhancing the translation of mRNA transcripts encoding immune-suppressive molecules, such as PD-L1, YTHDF1 facilitates immune escape by enabling liver cancer cells to evade immune detection and destruction. This translational control is particularly critical in liver cancer, where the tumor microenvironment is frequently immunosuppressive. The increased translation of PD-L1 and other immunosuppressive factors driven by YTHDF1 contributes to the establishment of an immune-resistant environment, hindering effective anti-tumor immune responses. Through its translational regulation of these key immune molecules, YTHDF1 plays a central role in promoting immune evasion and tumor progression. In addition to its role in immune checkpoint regulation, YTHDF1 also influences immune cell infiltration within the tumor microenvironment by modulating the translation of genes involved in immune cell recruitment and activity. YTHDF1 affects the recruitment and activation of immune cells such as macrophages, NK cells, and CD8^+^ T cells, thereby altering the immune cell composition within the tumor. Through its impact on the translation of mRNAs encoding factors involved in immune cell trafficking, YTHDF1 can promote the accumulation of immune-suppressive cells like M2 macrophages and Tregs, while simultaneously inhibiting the activation of anti-tumor T cells. This disruption of immune cell dynamics reduces the ability of the immune system to identify and eliminate tumor cells. Elevated expression of YTHDF1 in liver cancer cells inhibits immune cell activation and proliferation by regulating the translation of immune-related transcripts, ultimately fostering an immune-suppressive environment that allows tumor cells to escape immune surveillance (45). Despite the emerging evidence highlighting the role of YTHDF1 in immune modulation and tumor progression, research on its precise mechanisms in liver cancer remains in the early stages. Understanding how YTHDF1 influences both mRNA translation and immune responses is crucial for developing targeted therapies. Given its significant role in immune evasion, YTHDF1 presents a promising target for immunotherapy, particularly for overcoming resistance to immune checkpoint inhibitors. By inhibiting YTHDF1, it may be possible to reverse immune suppression within the tumor microenvironment, enhance immune cell activation, and improve the efficacy of immunotherapies. Targeting YTHDF1 could increase the susceptibility of liver cancer to immune checkpoint blockade, thus improving survival outcomes for patients. Nevertheless, additional studies are required to comprehensively uncover the molecular mechanisms through which YTHDF1 regulates translation and to better understand its broader impact on liver cancer biology. This will lay the foundation for developing innovative therapeutic strategies (36, 46).

YTHDF2, an important m^6^A reader protein, primarily facilitates mRNA degradation by binding to transcripts modified by m^6^A. This process is crucial for regulating the expression of immune-related genes in liver cancer cells. Studies have shown that YTHDF2 influences the stability of mRNAs encoding immune-regulatory factors, thereby affecting the protein levels of these factors (47). For example, YTHDF2 can accelerate the degradation of mRNA transcripts for immune-suppressive molecules, such as PD-L1, which reduces immune suppression and enhances the immune system’s ability to target and eliminate liver cancer cells. By modulating the translation of these immune-related genes, YTHDF2 plays a pivotal role in controlling immune responses within the liver cancer microenvironment, allowing for more effective immune surveillance (48). In addition to regulating mRNA degradation, YTHDF2 influences the expression of surface antigens on liver cancer cells, thereby increasing the tumor’s visibility to the immune system. By promoting the degradation of mRNAs encoding certain immune evasion molecules and antigens, YTHDF2 may enhance tumor cell recognition by immune cells and improve their susceptibility to immune-mediated clearance. Moreover, YTHDF2 has been implicated in modulating immune cell function within the tumor microenvironment. Through its effects on the translation of immune-related transcripts, YTHDF2 can influence the infiltration and distribution of various immune cells, such as macrophages, NK cells, and CD8^+^ T cells. This regulation of immune cell dynamics can enhance the effectiveness of immunotherapies, particularly immune checkpoint inhibitors, by reshaping the tumor immune microenvironment and improving the immune response to liver cancer (3). Despite these promising findings, the precise mechanisms by which YTHDF2 regulates translation and modulates immune responses in liver cancer remain to be fully elucidated. Further research is needed to clarify how YTHDF2 orchestrates the translation of specific immune-related mRNAs and its broader role in liver cancer immunotherapy. Such investigations may identify novel therapeutic targets and provide new strategies for enhancing the efficacy of current immunotherapies. Ultimately, targeting YTHDF2 could serve as a promising approach to improve treatment outcomes and survival rates for patients with liver cancer, offering a more effective means of overcoming immune suppression and enhancing immune cell-mediated tumor clearance.

As a key m^6^A reader protein, YTHDC1 plays a crucial role in regulating the expression and function of immune-related genes in liver cancer. YTHDC1 specifically recognizes and binds to mRNA transcripts that are modified by m^6^A, influencing their processing and subsequent translation. One of its primary functions involves modulating the nuclear export of specific immune-related mRNAs, thereby controlling their expression levels in the cytoplasm. By promoting the transport of mRNAs encoding immune activation factors from the nucleus to the cytoplasm, YTHDC1 enhances the activation and function of immune cells. This, in turn, improves immune surveillance and cytotoxicity, enabling immune cells to more effectively target and eliminate liver cancer cells. Through this mechanism, YTHDC1 plays a pivotal role in enhancing the immune system’s capacity to respond to tumor cells, thereby contributing to the regulation of liver cancer immunotherapy (49). In addition to its role in immune activation, YTHDC1 may also influence the expression of immune evasion-related genes within liver cancer cells, thereby altering the tumor’s resistance to immune attack. By regulating the expression of genes involved in immune evasion, such as those encoding immune checkpoint molecules, YTHDC1 can modulate the immune microenvironment and affect the tumor’s susceptibility to immune-mediated destruction. This dual function of YTHDC1 in both immune activation and immune evasion makes it a critical regulator of liver cancer progression and response to immunotherapy. Despite these promising findings, the precise molecular mechanisms through which YTHDC1 regulates liver cancer immunotherapy remain insufficiently understood and require further in-depth investigation. Further studies are needed to elucidate how YTHDC1 regulates immune-related mRNA trafficking and translation in liver cancer cells, as well as its broader impact on immune responses within the tumor microenvironment. Such research is essential for identifying potential therapeutic targets and developing more effective strategies for liver cancer immunotherapy (50).

YTHDC2 is an important m^6^A reader protein. Although its specific mechanism in liver cancer immunotherapy has not been fully clarified, existing research suggests it may play a crucial role. Studies indicate that YTHDC2 might interact with m^6^A-modified mRNA, influencing the expression and regulation of related genes, thereby indirectly participating in the modulation of liver cancer immune responses (51). It may affect the expression of genes associated with immune signaling and immune cell infiltration within liver cancer cells, altering the tumor microenvironment and, consequently, influencing the effectiveness of immunotherapy. Some studies suggest that YTHDC2 might work synergistically with other immune-regulatory factors to co-regulate the liver cancer cells’ response to the immune system (52). For example, it may be involved in regulating the expression of immune checkpoint molecules, impacting the interaction between immune cells and liver cancer cells, and thus affecting the sensitivity to immunotherapy. However, the role of YTHDC2 in liver cancer immunotherapy still requires more in-depth research to clarify its specific mechanisms and clinical significance. Future exploration of YTHDC2 may provide new insights and targets for liver cancer immunotherapy, offering new opportunities to improve treatment outcomes and prognosis for patients.

Other m^6^A methylation readers, such as YTHDF3 and IGF2BP1/2/3, are closely linked to immune therapy in liver cancer. YTHDF3 has significant regulatory functions, as it precisely controls the translation efficiency of immune-related mRNA, thereby profoundly influencing the complex and delicate interactions between immune cells and liver cancer cells (53). On the other hand, IGF2BP1/2/3 stabilize specific m^6^A-modified mRNA, enabling fine-tuned regulation of immune-related gene expression, which in turn indirectly affects the overall outcome of liver cancer immunotherapy (14). These reader proteins work synergistically, contributing to the formation of a complex regulatory network. They play key roles in influencing immune responses in liver cancer cells, and significantly affect the sensitivity and effectiveness of immunotherapy. However, it is important to note that while we have gained some understanding of their roles, the specific mechanisms through which each of these proteins functions still require further in-depth and comprehensive investigation. Only by clarifying these mechanisms can we provide more practical and feasible possibilities for optimizing liver cancer immunotherapy strategies, ultimately offering more hopeful and effective treatment options for liver cancer patients.

### 3.3 The role of m^6^A methylation erasers in liver cancer immunotherapy

The m^6^A demethylase FTO plays a pivotal role in liver immune therapy, acting as a crucial epigenetic regulator that profoundly impacts immune processes in the liver through the demethylation of m^6^A modifications on RNA molecules. In the context of liver immune cells, FTO is integral to the regulation of macrophage function. It induces a shift in macrophage polarization, facilitating the transition from the pro-inflammatory M1 phenotype to the anti-inflammatory M2 phenotype within an inflammatory environment. This shift not only alleviates liver inflammation but also creates a favorable microenvironment conducive to tissue repair (22). Additionally, FTO exerts significant regulatory control over T cell activation, proliferation, and differentiation. By modulating the expression of T cell receptor-related genes, FTO can precisely govern T cell immune responses, ensuring their proper functioning and contributing to the maintenance of liver immune homeostasis. At the molecular level, the regulatory influence of FTO on immune-related gene expression is a key determinant in the modulation of liver immune responses, underscoring its critical role in liver immune therapeutic strategies. By specifically demethylating m6A marks on the mRNA of these genes, FTO influences their expression profiles, thereby affecting key processes such as immune responses, inflammation regulation, and cell-to-cell communication. This regulatory action has profound implications for the stability and dynamics of the liver immune microenvironment. As a complex network of cellular and molecular interactions, the liver immune microenvironment is finely tuned by FTO, which regulates the interactions between liver cells, immune cells, and components of the extracellular matrix, thereby maintaining immune homeostasis (54). Moreover, FTO can modulate cytokine secretion, influencing intercellular signaling pathways and thereby precisely adjusting the liver’s immune status to respond effectively to a range of pathogenic challenges (55). In summary, FTO, as an m^6^A demethylase, plays a multifaceted and indispensable role in liver immune therapy, offering novel insights into liver immune mechanisms. Its potential as a therapeutic target provides new opportunities for the development of innovative strategies aimed at enhancing the efficacy of liver disease treatments.

ALKBH5, an m^6^A demethylase, exerts critical regulatory control over RNA stability by removing m6A modifications, thereby modulating fundamental cellular processes such as proliferation, apoptosis, and metastasis. In the context of liver cancer, ALKBH5 has been implicated in promoting tumor progression by enhancing immune evasion mechanisms within the tumor microenvironment. Elevated expression of ALKBH5 in liver cancer patients is strongly associated with the upregulation of immune-suppressive markers, such as PD-L1, which facilitate immune escape and contribute to tumor progression by impeding effective immune surveillance. Moreover, ALKBH5 influences the expression of a range of immune-related genes, thereby fostering a suppressive microenvironment that promotes tumor survival and metastasis. These findings underscore the multifaceted role of ALKBH5 in both regulating tumor cell behavior and shaping the immune landscape within the liver cancer microenvironment, positioning it as a potential therapeutic target in liver cancer management (56). In the realm of liver cancer immunotherapy, ALKBH5 plays a crucial modulatory role. Inhibition of ALKBH5 has been shown to enhance T cell infiltration and activation within the tumor, thereby strengthening the anti-tumor immune response. This effect is primarily mediated through the upregulation of m^6^A modifications, which in turn increase the expression of immune-related genes, including cytokines and chemokines, essential for facilitating immune recognition and tumor cell elimination. Furthermore, high ALKBH5 expression correlates with poor clinical outcomes in patients undergoing immune checkpoint inhibitor (ICI) therapy, suggesting that ALKBH5 may act as a modulator of immune checkpoint resistance. In this context, the inhibition of ALKBH5 could serve as a strategy to restore immune checkpoint efficacy, particularly in overcoming resistance to PD-1/PD-L1 blockade. Additionally, ALKBH5 has been implicated in regulating immune responses in the liver cancer-associated hepatitis microenvironment, thus offering new avenues for enhancing the effectiveness of liver cancer immunotherapy. Transcriptomic analyses have demonstrated that ALKBH5 modulates both the intra- and extracellular liver cancer cell microenvironment, influencing macrophage polarization and hepatic cell transformation, thereby contributing to immune evasion in liver cancer (56). Given the complexity of its roles, future research should prioritize a deeper understanding of ALKBH5’s molecular mechanisms, particularly its downstream signaling pathways and functional variations across liver cancer subtypes. Moreover, exploring combination therapies involving ALKBH5 inhibition, small molecules, and gene editing technologies may enhance treatment efficacy and improve patient outcomes. ALKBH5 regulates liver cancer immunotherapy by modulating RNA demethylation, impacting tumor biology and immune responses. Targeting ALKBH5 offers a promising approach to enhance immunotherapy efficacy. Further research into its mechanisms, combined with clinical data and innovative strategies, could significantly improve treatment outcomes for liver cancer patients (57).

## 4 Discussion

M^6^A is one of the most common internal modifications in mRNA. It plays a crucial role in a variety of biological processes by influencing RNA transcription, splicing, nuclear export, translation, and stability. In recent years, the critical role of m^6^A modifications in HCC has gradually been unveiled, particularly in the regulation of the immune microenvironment and its impact on immunotherapy. Studies have shown that m^6^A modifications significantly affect the immune evasion capacity of tumors and their response to immunotherapy by regulating the expression of immune-related genes, stabilizing immune suppressive molecules, and modulating the secretion of inflammatory factors.

The immune microenvironment of HCC is composed of a complex network of tumor cells, immune cells, and stromal elements, all of which are interdependent. m6A modification exerts profound effects on immune cell functions and the composition of the immune microenvironment. It regulates the expression of key genes involved in immune responses and modulates the interaction between immune cells and tumor cells, which directly impacts tumor immune evasion and therapy resistance. One of the key aspects of m^6^A’s role in the immune microenvironment is its ability to modulate immune cell infiltration and polarization. M^6^A modification influences the recruitment of immune cells to the tumor site, particularly immune-suppressive cells such as macrophages and Tregs. It also regulates macrophage polarization, promoting the transition of macrophages from pro-inflammatory M1 to anti-inflammatory M2 phenotypes, thus enhancing the immunosuppressive environment within the tumor. These changes in immune cell dynamics can lead to a more immunosuppressive tumor microenvironment, ultimately undermining the efficacy of immunotherapy.

Furthermore, m^6^A affects the translation and stability of various cytokines and chemokines, which play pivotal roles in immune cell recruitment and activation. By regulating the expression of these molecules, m^6^A controls the immune balance and can shift it toward an immunosuppressive state that favors tumor progression. Studies have demonstrated that m^6^A modification of immune-related mRNAs such as IL-6, TNF-α, and various chemokines is critical for maintaining immune homeostasis in the liver and for shaping the immune response in HCC. This regulation of immune signaling not only influences tumor cell behaviors but also directly impacts the effectiveness of immune therapies by modulating the immune microenvironment. The understanding of m^6^A modification in regulating immune responses has opened up new possibilities for enhancing the effectiveness of immunotherapy in HCC. Immunotherapy, especially ICIs, has revolutionized the treatment of several cancers, but its success in HCC has been limited, partly due to the immune evasion mechanisms employed by tumor cells. m6A modification plays a crucial role in regulating the expression of immune checkpoint molecules such as PD-L1, as well as immune-related genes, making it a promising target for enhancing the efficacy of immunotherapy. Targeting m^6^A “writers” or “erasers” could offer novel therapeutic strategies to overcome immune resistance in HCC. For instance, inhibiting METTL3 or METTL14, which increase PD-L1 expression on tumor cells, could reduce immune escape and restore T-cell activity, thus enhancing the response to immune checkpoint blockade. On the other hand, targeting m^6^A “erasers” such as ALKBH5 or FTO, which regulate immune checkpoint molecule expression by demethylating m6A modifications, could suppress immune suppression and promote anti-tumor immune responses. Additionally, combining m^6^A-targeted therapies with existing immunotherapies, such as ICIs, may provide synergistic effects that improve therapeutic outcomes in HCC patients.

However, the precise mechanisms through which m^6^A influences immune responses and its impact on different HCC subtypes remain to be fully elucidated. Since HCC is a heterogeneous disease, it is important to explore how m^6^A modifies immune evasion mechanisms across different patient populations and tumor subtypes. Further research into m^6^A’s role in immune modulation and tumor microenvironment remodeling, as well as its interaction with other therapeutic modalities, is needed to develop personalized treatment strategies. Moreover, clinical trials targeting m^6^A modification in combination with ICIs or other immunotherapies will be essential to validate the therapeutic potential of m^6^A modulation in improving HCC immunotherapy outcomes.

M^6^A modification plays a critical role in the immune evasion mechanisms of HCC and significantly influences the tumor immune microenvironment. By modulating immune checkpoint molecule expression, cytokine production, and immune cell polarization, m^6^A contributes to the resistance of HCC to immune therapies. Targeting m^6^A “writers” and “erasers” offers a promising avenue for enhancing the effectiveness of immunotherapy in HCC. As our understanding of m^6^A’s role in immune regulation deepens, it is expected that new therapeutic strategies targeting m^6^A modification will emerge, potentially transforming the landscape of liver cancer immunotherapy.

## 5 Conclusion

Despite significant progress in the research on m^6^A modifications in liver cancer immunotherapy in recent years, many critical issues remain unresolved. For instance, the dynamic regulatory mechanisms of m^6^A modifications in liver cancer and its immune microenvironment, as well as their specific functions in different patient populations, are still unclear. Moreover, developing highly specific and low-toxicity small molecule inhibitors targeting m^6^A regulatory proteins could provide more precise and efficient treatment options for liver cancer patients. A deeper exploration of the molecular mechanisms of m^6^A modifications, and incorporating them into immunotherapy strategies, holds the potential to offer new perspectives in overcoming the current challenges in liver cancer immunotherapy and further promote the advancement of precision medicine (**Table 1**).

**Table 1:** Molecular mechanism of m^6^A methylation involved in immune regulation of liver cancer.

m6A Regulator	Target	Impact on immune evasion mechanisms	Mechanism
METTL3 (58)	PD-L1, CTLA-4, SOCS3	Enhances expression of immune checkpoints, suppresses T cell activity, promotes immune evasion.	METTL3 catalyzes m6A modification, regulating the expression of immune checkpoint molecules.
METTL14 (59)	TGF-β, IL-6, CXCL8	Increases secretion of immune suppressive cytokines like TGF-β and IL-6, inhibiting anti-tumor immunity.	METTL14 regulates cytokine expression, affecting the immune microenvironment and enhancing immune suppression.
FTO (54)	PD-L1, CTLA-4, IL-10	Inhibits immune responses through demethylation, promoting tumor immune escape.	FTO removes m6A modifications, regulating immune-related gene expression and enhancing immune evasion.
ALKBH5 (56)	TGF-β, JAK2/STAT3	Activates immune evasion pathways, suppressing immune cell functions via JAK-STAT signaling.	ALKBH5 removes m6A modifications, enhancing TGF-β signaling and promoting immune evasion.
YTHDF1/2/3 (60)	PD-L1, IFN-γ, IL-2	Modulates immune evasion factors, suppresses T cell functions and cytokine production.	The YTHDF family recognizes m6A modifications and regulates the expression of immune-related genes, promoting immune evasion.
IGF2BP1/2/3 (61)	IL-6, CXCL8, VEGF	Stabilizes immune suppressive factors like IL-6 and CXCL8, suppressing immune responses.	The IGF2BP family binds to m6A-modified mRNAs, stabilizing immune-related factors and enhancing immune evasion.
miR-21 (62)	SOCS1, PDCD4	miR-21, a downstream non-coding RNA regulated by m6A, modulates immune evasion-related molecules.	Inhibits the expression of SOCS1 and PDCD4, enhancing immune evasion mechanisms, promoting liver cancer cell growth.
WTAP (63)	PD-L1, SOCS1, IL-10	Regulates immune checkpoint expression and stabilizes immune suppressive cytokines like IL-10, promoting immune evasion.	WTAP acts as a cofactor for METTL3/METTL14 complex, promoting m6A modification of immune regulators.
KIAA1429 (64)	TGF-β, TNF-α	Enhances immune suppression via regulation of TGF-β and TNF-α pathways, promoting immune evasion.	KIAA1429 is an m6A methyltransferase that affects mRNA stability and translation.
